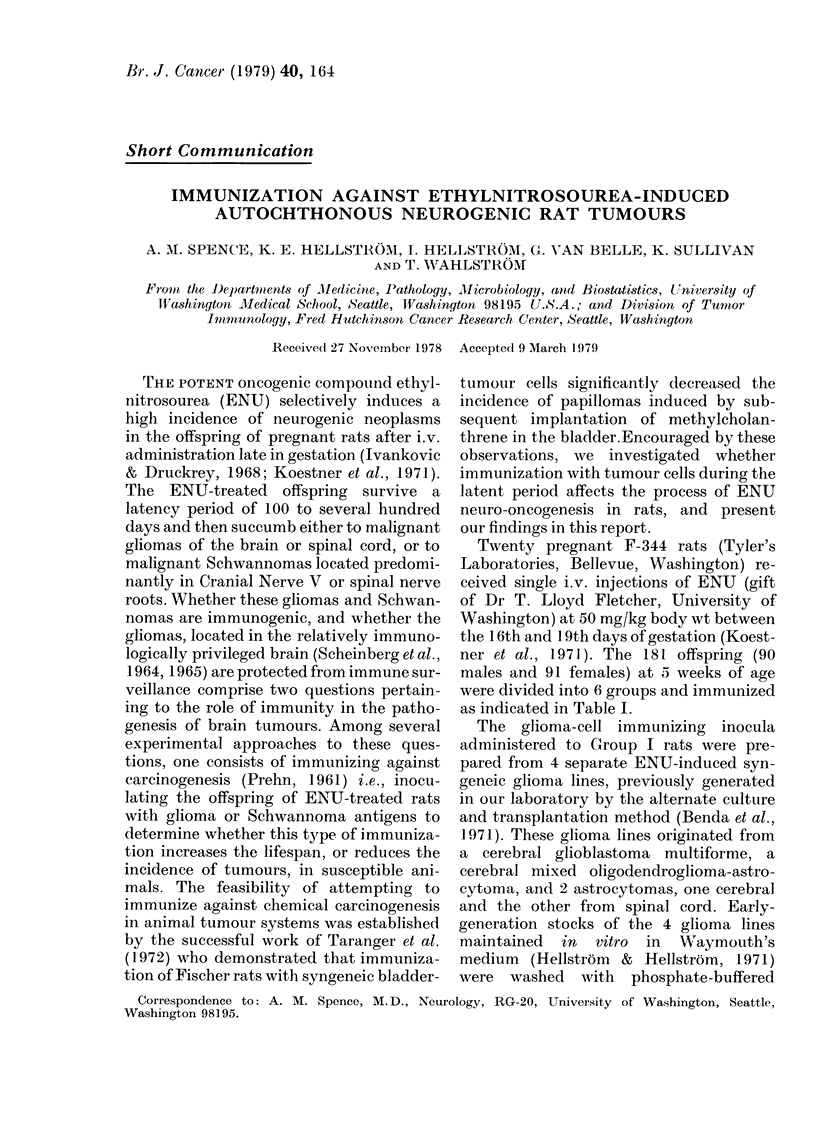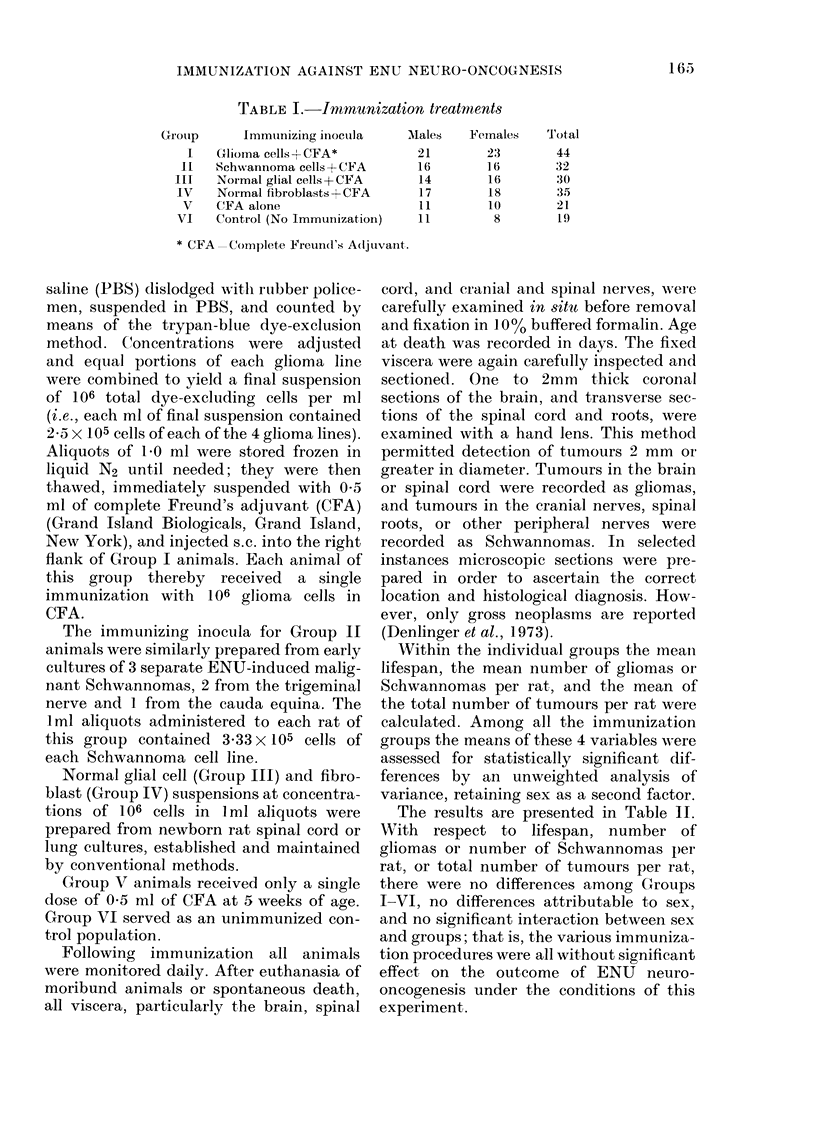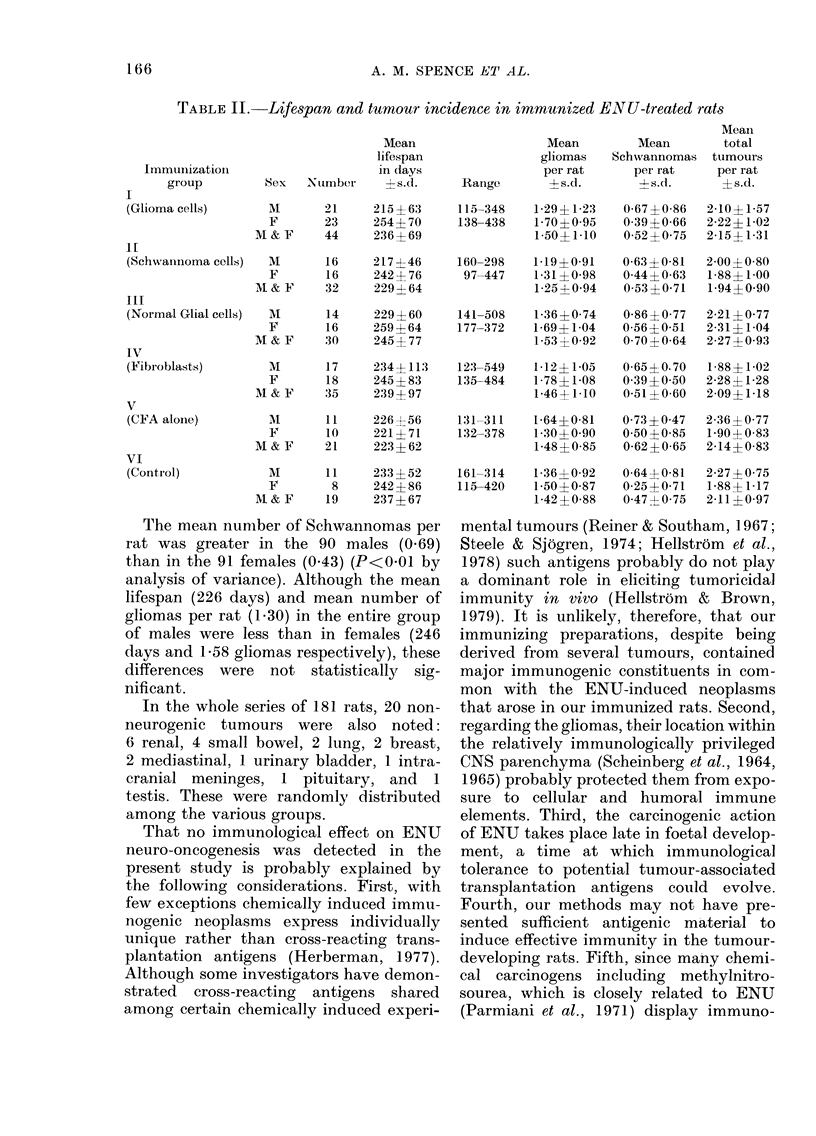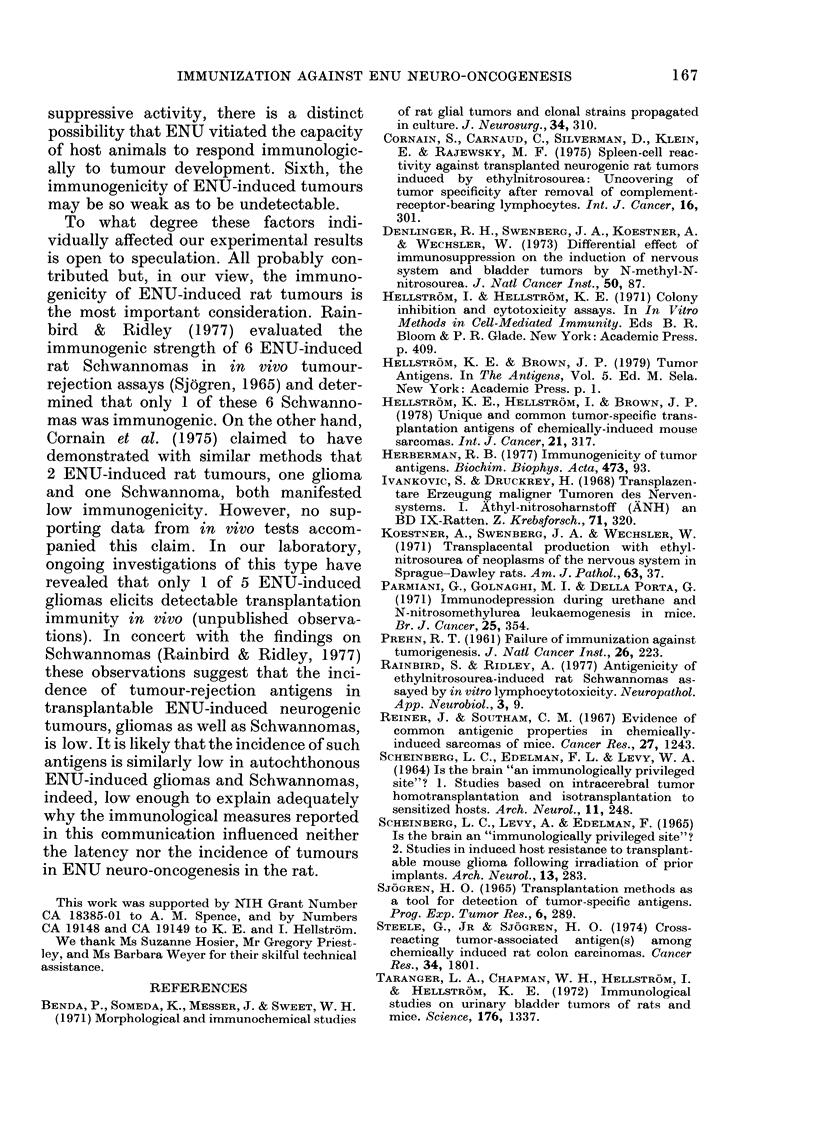# Immunization against ethylnitrosourea-induced autochthonous neurogenic rat tumours.

**DOI:** 10.1038/bjc.1979.154

**Published:** 1979-07

**Authors:** A. M. Spence, K. E. Hellström, I. Hellström, G. Van Belle, K. Sullivan, T. Wahlström


*Br. J. Cancer* (1979) **40,** 164

***Short Communication***

### IMMUNIZATION AGAINST ETHYLNITROSOUREA-INDUCED AUTOCHTHONOUS NEUROGENIC RAT TUMOURS

A. M. SPENCE, K. E. HELLSTRÖM, I. HELLSTRÖM, G. VAN BELLE, K. SULLIVAN AND T. WAHLSTRÖM

*From the Departments of Medicine, Pathology, Microbiology, and Biostatistics, University of Washington Medical School, Seattle, Washington* 98195 *U.S.A.; and Division of Tumor Immunology, Fred Hutchinson Cancer Research Center, Seattle, Washington*

Received 27 November 1978 Accepted 9 March 1979

THE POTENT oncogenic compound ethylnitrosourea (ENU) selectively induces a high incidence of neurogenic neoplasms in the offspring of pregnant rats after i.v. administration late in gestation (Ivankovic & Druckrey, 1968; Koestner *et al.*, 1971). The ENU-treated offspring survive a latency period of 100 to several hundred days and then succumb either to malignant gliomas of the brain or spinal cord, or to malignant Schwannomas located predominantly in Cranial Nerve V or spinal nerve roots. Whether these gliomas and Schwannomas are immunogenic, and whether the gliomas, located in the relatively immunologically privileged brain (Scheinberg *et al.*, 1964, 1965) are protected from immune surveillance comprise two questions pertaining to the role of immunity in the pathogenesis of brain tumours. Among several experimental approaches to these questions, one consists of immunizing against carcinogenesis (Prehn, 1961) *i.e.*, inoculating the offspring of ENU-treated rats with glioma or Schwannoma antigens to determine whether this type of immunization increases the lifespan, or reduces the incidence of tumours, in susceptible animals. The feasibility of attempting to immunize against chemical carcinogenesis in animal tumour systems was established by the successful work of Taranger *et al.* (1972) who demonstrated that immunization of Fischer rats with syngeneic bladder-tumour cells significantly decreased the incidence of papillomas induced by subsequent implantation of methylcholanthrene in the bladder.Encouraged by these observations, we investigated whether immunization with tumour cells during the latent period affects the process of ENU neuro-oncogenesis in rats, and present our findings in this report.

Twenty pregnant F-344 rats (Tyler's Laboratories, Bellevue, Washington) received single i.v. injections of ENU (gift of Dr T. Lloyd Fletcher, University of Washington) at 50 mg/kg body wt between the 16th and 19th days of gestation (Koestner *et al.*, 1971). The 181 offspring (90 males and 91 females) at 5 weeks of age were divided into 6 groups and immunized as indicated in Table I.

The glioma-cell immunizing inocula administered to Group I rats were prepared from 4 separate ENU-induced syngeneic glioma lines, previously generated in our laboratory by the alternate culture and transplantation method (Benda *et al.*, 1971). These glioma lines originated from a cerebral glioblastoma multiforme, a cerebral mixed oligodendroglioma-astrocytoma, and 2 astrocytomas, one cerebral and the other from spinal cord. Early-generation stocks of the 4 glioma lines maintained *in vitro* in Waymouth's medium (Hellström & Hellström, 1971) were washed with phosphate-buffered

Correspondence to: A. M. Spence, M.D., Neurology, RG-20, University of Washington, Seattle, Washington 98195.

TABLE I.—*Immunization treatments*

| Group | Immunizing inocula | Males | Females | Total |
|---|---|---|---|---|
| I | Glioma cells+CFA* | 21 | 23 | 44 |
| II | Schwannoma cells+CFA | 16 | 16 | 32 |
| III | Normal glial cells+CFA | 14 | 16 | 30 |
| IV | Normal fibroblasts+CFA | 17 | 18 | 35 |
| V | CFA alone | 11 | 10 | 21 |
| VI | Control (No Immunization) | 11 | 8 | 19 |

* CFA—Complete Freund's Adjuvant.

saline (PBS) dislodged with rubber policemen, suspended in PBS, and counted by means of the trypan-blue dye-exclusion method. Concentrations were adjusted and equal portions of each glioma line were combined to yield a final suspension of $10^6$ total dye-excluding cells per ml (*i.e.*, each ml of final suspension contained $2 \cdot 5 \times 10^5$ cells of each of the 4 glioma lines). Aliquots of 1·0 ml were stored frozen in liquid $N_2$ until needed; they were then thawed, immediately suspended with 0·5 ml of complete Freund's adjuvant (CFA) (Grand Island Biologicals, Grand Island, New York), and injected s.c. into the right flank of Group I animals. Each animal of this group thereby received a single immunization with $10^6$ glioma cells in CFA.

The immunizing inocula for Group II animals were similarly prepared from early cultures of 3 separate ENU-induced malignant Schwannomas, 2 from the trigeminal nerve and 1 from the cauda equina. The 1ml aliquots administered to each rat of this group contained $3 \cdot 33 \times 10^5$ cells of each Schwannoma cell line.

Normal glial cell (Group III) and fibroblast (Group IV) suspensions at concentrations of $10^6$ cells in 1ml aliquots were prepared from newborn rat spinal cord or lung cultures, established and maintained by conventional methods.

Group V animals received only a single dose of 0·5 ml of CFA at 5 weeks of age. Group VI served as an unimmunized control population.

Following immunization all animals were monitored daily. After euthanasia of moribund animals or spontaneous death, all viscera, particularly the brain, spinal cord, and cranial and spinal nerves, were carefully examined *in situ* before removal and fixation in 10% buffered formalin. Age at death was recorded in days. The fixed viscera were again carefully inspected and sectioned. One to 2mm thick coronal sections of the brain, and transverse sections of the spinal cord and roots, were examined with a hand lens. This method permitted detection of tumours 2 mm or greater in diameter. Tumours in the brain or spinal cord were recorded as gliomas, and tumours in the cranial nerves, spinal roots, or other peripheral nerves were recorded as Schwannomas. In selected instances microscopic sections were prepared in order to ascertain the correct location and histological diagnosis. However, only gross neoplasms are reported (Denlinger *et al.*, 1973).

Within the individual groups the mean lifespan, the mean number of gliomas or Schwannomas per rat, and the mean of the total number of tumours per rat were calculated. Among all the immunization groups the means of these 4 variables were assessed for statistically significant differences by an unweighted analysis of variance, retaining sex as a second factor.

The results are presented in Table II. With respect to lifespan, number of gliomas or number of Schwannomas per rat, or total number of tumours per rat, there were no differences among Groups I–VI, no differences attributable to sex, and no significant interaction between sex and groups; that is, the various immunization procedures were all without significant effect on the outcome of ENU neuro-oncogenesis under the conditions of this experiment.

TABLE II.—*Lifespan and tumour incidence in immunized ENU-treated rats*

| Immunization group | Sex | Number | Mean lifespan in days ±s.d. | Range | Mean gliomas per rat ±s.d. | Mean Schwannomas per rat ±s.d. | Mean total tumours per rat ±s.d. |
|---|---|---|---|---|---|---|---|
| I | | | | | | | |
| (Glioma cells) | M | 21 | 215±63 | 115–348 | 1·29±1·23 | 0·67±0·86 | 2·10±1·57 |
| | F | 23 | 254±70 | 138–438 | 1·70±0·95 | 0·39±0·66 | 2·22±1·02 |
| | M & F | 44 | 236±69 | | 1·50±1·10 | 0·52±0·75 | 2·15±1·31 |
| II | | | | | | | |
| (Schwannoma cells) | M | 16 | 217±46 | 160–298 | 1·19±0·91 | 0·63±0·81 | 2·00±0·80 |
| | F | 16 | 242±76 | 97–447 | 1·31±0·98 | 0·44±0·63 | 1·88±1·00 |
| | M & F | 32 | 229±64 | | 1·25±0·94 | 0·53±0·71 | 1·94±0·90 |
| III | | | | | | | |
| (Normal Glial cells) | M | 14 | 229±60 | 141–508 | 1·36±0·74 | 0·86±0·77 | 2·21±0·77 |
| | F | 16 | 259±64 | 177–372 | 1·69±1·04 | 0·56±0·51 | 2·31±1·04 |
| | M & F | 30 | 245±77 | | 1·53±0·92 | 0·70±0·64 | 2·27±0·93 |
| IV | | | | | | | |
| (Fibroblasts) | M | 17 | 234±113 | 123–549 | 1·12±1·05 | 0·65±0.70 | 1·88±1·02 |
| | F | 18 | 245±83 | 135–484 | 1·78±1·08 | 0·39±0·50 | 2·28±1·28 |
| | M & F | 35 | 239±97 | | 1·46±1·10 | 0·51±0·60 | 2·09±1·18 |
| V | | | | | | | |
| (CFA alone) | M | 11 | 226±56 | 131–311 | 1·64±0·81 | 0·73±0·47 | 2·36±0·77 |
| | F | 10 | 221±71 | 132–378 | 1·30±0·90 | 0·50±0·85 | 1·90±0·83 |
| | M & F | 21 | 223±62 | | 1·48±0·85 | 0·62±0·65 | 2·14±0·83 |
| VI | | | | | | | |
| (Control) | M | 11 | 233±52 | 161–314 | 1·36±0·92 | 0·64±0·81 | 2·27±0·75 |
| | F | 8 | 242±86 | 115–420 | 1·50±0·87 | 0·25±0·71 | 1·88±1·17 |
| | M & F | 19 | 237±67 | | 1·42±0·88 | 0·47±0·75 | 2·11±0·97 |

The mean number of Schwannomas per rat was greater in the 90 males (0·69) than in the 91 females (0·43) ($P<0·01$ by analysis of variance). Although the mean lifespan (226 days) and mean number of gliomas per rat (1·30) in the entire group of males were less than in females (246 days and 1·58 gliomas respectively), these differences were not statistically significant.

In the whole series of 181 rats, 20 non-neurogenic tumours were also noted: 6 renal, 4 small bowel, 2 lung, 2 breast, 2 mediastinal, 1 urinary bladder, 1 intracranial meninges, 1 pituitary, and 1 testis. These were randomly distributed among the various groups.

That no immunological effect on ENU neuro-oncogenesis was detected in the present study is probably explained by the following considerations. First, with few exceptions chemically induced immunogenic neoplasms express individually unique rather than cross-reacting transplantation antigens (Herberman, 1977). Although some investigators have demonstrated cross-reacting antigens shared among certain chemically induced experimental tumours (Reiner & Southam, 1967; Steele & Sjögren, 1974; Hellström *et al.*, 1978) such antigens probably do not play a dominant role in eliciting tumoricidal immunity *in vivo* (Hellström & Brown, 1979). It is unlikely, therefore, that our immunizing preparations, despite being derived from several tumours, contained major immunogenic constituents in common with the ENU-induced neoplasms that arose in our immunized rats. Second, regarding the gliomas, their location within the relatively immunologically privileged CNS parenchyma (Scheinberg *et al.*, 1964, 1965) probably protected them from exposure to cellular and humoral immune elements. Third, the carcinogenic action of ENU takes place late in foetal development, a time at which immunological tolerance to potential tumour-associated transplantation antigens could evolve. Fourth, our methods may not have presented sufficient antigenic material to induce effective immunity in the tumour-developing rats. Fifth, since many chemical carcinogens including methylnitrosourea, which is closely related to ENU (Parmiani *et al.*, 1971) display immunosuppressive activity, there is a distinct possibility that ENU vitiated the capacity of host animals to respond immunologically to tumour development. Sixth, the immunogenicity of ENU-induced tumours may be so weak as to be undetectable.

To what degree these factors individually affected our experimental results is open to speculation. All probably contributed but, in our view, the immunogenicity of ENU-induced rat tumours is the most important consideration. Rainbird & Ridley (1977) evaluated the immunogenic strength of 6 ENU-induced rat Schwannomas in *in vivo* tumour-rejection assays (Sjögren, 1965) and determined that only 1 of these 6 Schwannomas was immunogenic. On the other hand, Cornain *et al.* (1975) claimed to have demonstrated with similar methods that 2 ENU-induced rat tumours, one glioma and one Schwannoma, both manifested low immunogenicity. However, no supporting data from *in vivo* tests accompanied this claim. In our laboratory, ongoing investigations of this type have revealed that only 1 of 5 ENU-induced gliomas elicits detectable transplantation immunity *in vivo* (unpublished observations). In concert with the findings on Schwannomas (Rainbird & Ridley, 1977) these observations suggest that the incidence of tumour-rejection antigens in transplantable ENU-induced neurogenic tumours, gliomas as well as Schwannomas, is low. It is likely that the incidence of such antigens is similarly low in autochthonous ENU-induced gliomas and Schwannomas, indeed, low enough to explain adequately why the immunological measures reported in this communication influenced neither the latency nor the incidence of tumours in ENU neuro-oncogenesis in the rat.

This work was supported by NIH Grant Number CA 18385-01 to A. M. Spence, and by Numbers CA 19148 and CA 19149 to K. E. and I. Hellström.

We thank Ms Suzanne Hosier, Mr Gregory Priestley, and Ms Barbara Weyer for their skilful technical assistance.

### REFERENCES

Benda, P., Someda, K., Messer, J. & Sweet, W. H. (1971) Morphological and immunochemical studies of rat glial tumors and clonal strains propagated in culture. *J. Neurosurg.*, **34**, 310.

Cornain, S., Carnaud, C., Silverman, D., Klein, E. & Rajewsky, M. F. (1975) Spleen-cell reactivity against transplanted neurogenic rat tumors induced by ethylnitrosourea: Uncovering of tumor specificity after removal of complement-receptor-bearing lymphocytes. *Int. J. Cancer*, **16**, 301.

Denlinger, R. H., Swenberg, J. A., Koestner, A. & Wechsler, W. (1973) Differential effect of immunosuppression on the induction of nervous system and bladder tumors by N-methyl-N-nitrosourea. *J. Natl Cancer Inst.*, **50**, 87.

Hellström, I. & Hellström, K. E. (1971) Colony inhibition and cytotoxicity assays. In *In Vitro Methods in Cell-Mediated Immunity.* Eds B. R. Bloom & P. R. Glade. New York: Academic Press. p. 409.

Hellström, K. E. & Brown, J. P. (1979) Tumor Antigens. In *The Antigens*, Vol. 5. Ed. M. Sela. New York: Academic Press. p. 1.

Hellström, K. E., Hellström, I. & Brown, J. P. (1978) Unique and common tumor-specific transplantation antigens of chemically-induced mouse sarcomas. *Int. J. Cancer*, **21**, 317.

Herberman, R. B. (1977) Immunogenicity of tumor antigens. *Biochim. Biophys. Acta*, **473**, 93.

Ivankovic, S. & Druckrey, H. (1968) Transplazentare Erzeugung maligner Tumoren des Nervensystems. I. Äthyl-nitrosoharnstoff (ÄNH) an BD IX-Ratten. *Z. Krebsforsch.*, **71**, 320.

Koestner, A., Swenberg, J. A. & Wechsler, W. (1971) Transplacental production with ethylnitrosourea of neoplasms of the nervous system in Sprague–Dawley rats. *Am. J. Pathol.*, **63**, 37.

Parmiani, G., Golnaghi, M. I. & Della Porta, G. (1971) Immunodepression during urethane and N-nitrosomethylurea leukaemogenesis in mice. *Br. J. Cancer*, **25**, 354.

Prehn, R. T. (1961) Failure of immunization against tumorigenesis. *J. Natl Cancer Inst.*, **26**, 223.

Rainbird, S. & Ridley, A. (1977) Antigenicity of ethylnitrosourea-induced rat Schwannomas assayed by *in vitro* lymphocytotoxicity. *Neuropathol. App. Neurobiol.*, **3**, 9.

Reiner, J. & Southam, C. M. (1967) Evidence of common antigenic properties in chemically-induced sarcomas of mice. *Cancer Res.*, **27**, 1243.

Scheinberg, L. C., Edelman, F. L. & Levy, W. A. (1964) Is the brain "an immunologically privileged site"? 1. Studies based on intracerebral tumor homotransplantation and isotransplantation to sensitized hosts. *Arch. Neurol.*, **11**, 248.

Scheinberg, L. C., Levy, A. & Edelman, F. (1965) Is the brain an "immunologically privileged site"? 2. Studies in induced host resistance to transplantable mouse glioma following irradiation of prior implants. *Arch. Neurol.*, **13**, 283.

Sjögren, H. O. (1965) Transplantation methods as a tool for detection of tumor-specific antigens. *Prog. Exp. Tumor Res.*, **6**, 289.

Steele, G., Jr & Sjögren, H. O. (1974) Cross-reacting tumor-associated antigen(s) among chemically induced rat colon carcinomas. *Cancer Res.*, **34**, 1801.

Taranger, L. A., Chapman, W. H., Hellström, I. & Hellström, K. E. (1972) Immunological studies on urinary bladder tumors of rats and mice. *Science*, **176**, 1337.